# Mortality related to primary bariatric surgery in England

**DOI:** 10.1002/bjs5.20

**Published:** 2017-10-26

**Authors:** M. Alam, S. Bhanderi, J. H. Matthews, D. McNulty, D. Pagano, P. Small, R. Singhal, R. Welbourn

**Affiliations:** ^1^ Upper Gastrointestinal and Bariatric Unit, Heartlands Hospital, Heart of England NHS Foundation Trust Birmingham UK; ^2^ Health Informatics University Hospital Birmingham NHS Foundation Trust Birmingham UK; ^3^ Quality and Outcomes Research Unit University Hospital Birmingham NHS Foundation Trust Birmingham UK; ^4^ Directorate of General Surgery, City Hospitals Sunderland NHS Foundation Trust Sunderland UK; ^5^ Department of Upper Gastrointestinal and Bariatric Surgery Musgrove Park Hospital Taunton UK

## Abstract

**Background:**

Bariatric surgery is an accepted treatment option for severe obesity. Previous analysis of the independently collected Hospital Episode Statistics (HES) data for outcomes after bariatric surgery demonstrated a 30‐day postoperative mortality rate of 0·3 per cent in the English National Health Service (NHS). However, there have been no published mortality data for bariatric procedures performed since 2008. This study aimed to assess mortality related to bariatric surgery in England from 2009.

**Methods:**

HES data were used to identify all patients who had primary bariatric surgery from 2009 to 2016. Clinical codes were used selectively to identify all primary bariatric procedures but exclude revision or conversion procedures and operations for malignant or other benign disease. The primary outcome measures were HES in‐hospital and Office for National Statistics (ONS) 30‐day mortality after discharge.

**Results:**

A total of 41 241 primary bariatric procedures were carried out in the NHS between 2009 and 2016, with 29 in‐hospital deaths (0·07 per cent). The 30‐day mortality rate after discharge was 0·08 per cent (32 of 41 241). Both the in‐hospital and 30‐day mortality rates after discharge demonstrated a downward trend over the study period.

**Conclusion:**

Overall in‐hospital and 30‐day mortality rates remain very low after primary bariatric surgery. An increased uptake of bariatric surgery within the English NHS has been safe.

## Introduction

In the UK, one in four adults and approximately one in five children are now obese, with a BMI of 30 kg/m^2^ or more^1^. For patients with severe and complex obesity (BMI at least 40 kg/m^2^ or 30 kg/m^2^ or above with obesity‐related disease), bariatric surgery has been shown to be significantly better than intensive lifestyle intervention at reducing weight and improving obesity‐related disease^2^ ^3^. The National Institute of Health and Care Excellence (NICE) recommends bariatric surgery as a treatment option for patients unable to lose weight by other means^4^. Although at least 5 per cent of the adult population in England exceeds the BMI thresholds for bariatric surgery, the uptake of National Health Service (NHS)‐funded surgery is much lower than in equivalent European countries^5^, ^6^, ^7^. The perceived risks of surgery may be one of the factors contributing to this.

Previous Hospital Episode Statistics (HES) analysis of the outcomes following bariatric surgery in England demonstrated a 30‐day postoperative mortality rate of 0·3 per cent for patients operated on between 2000 and 2008^8^. HES data are an independently collected, national data resource, containing diagnostic, procedural and in‐hospital outcome data for all admissions, outpatient and emergency department attendances in NHS trusts in England^9^. It is the only data source that facilitates mortality analysis throughout the English NHS as a whole. The Health and Social Care Information Centre (HSCIC) (now NHS Digital) publishes procedure rates for bariatric surgery based on a selection of HES procedure codes in the absence of specific codes for most types of bariatric surgery^10^. The lack of specific bariatric procedure codes raises the possibility that the selection used for previous analysis may not have captured all primary bariatric procedures, and may have included some non‐bariatric procedures, with inaccurate outcome reporting. No mortality data related to these procedures have been published since 2008, despite an increase in rates of surgery. This study aimed to assess mortality rates after bariatric surgery using an updated set of HES codes that reflect current bariatric surgical practice in the English NHS.

## Methods

### Data sources

Data on 30‐day mortality after discharge, annual procedural counts (total and operation subtypes) and in‐hospital mortality rates via an existing linkage between the HES database and the Office for National Statistics (ONS) births, marriages and deaths register were extracted^11^. Patients are assigned a single primary diagnostic code taken from the ICD‐10. Procedural codes for interventions are assigned from the relevant OPCS version. Patient demographics were not extracted from the HES database. HES does not include BMI, and no data were extracted regarding co‐morbidities as HES reporting of them may not be accurate.

### Inclusion and exclusion criteria

All NHS patients in England who underwent a primary, elective bariatric procedure between April 2009 and March 2016 were included. HES data include private patients treated at NHS hospitals and NHS patients treated at private hospitals; however, private patients were excluded from the present study. Secondary procedures were filtered using two mechanisms. The choice of OPCS codes eliminated secondary procedures with the description ‘Revision’ or ‘Conversion’. A second filter eliminated any procedure performed 1–30 days after a previous procedure. Secondary procedures involving conversion of a previous primary procedure to a new procedure (such as conversion of a gastric band to gastric bypass) were not included. Surgery involving revision of the anatomy of a primary bariatric procedure owing to therapeutic failure or early/late complications of surgery was also excluded.

Patients were identified from the HES data set using a combination of the principal reason for admission (ICD‐10 diagnostic code E66 – Obesity) and the procedures used for treatment (OPCS code). A procedure was assumed to have been elective when the primary diagnostic was E66. The list of OPCS codes selected was finalized from a list of 80 codes prevalent in the existing literature and publications from the HSCIC^6^ ^8^, ^12^ ^13^. Each code, code combinations and associated procedure descriptors were analysed individually, and cross‐referenced with the descriptions of bariatric procedures listed in the National Bariatric Registry (NBSR) reports^6^ ^12^. Two bariatric surgeons chose a list of codes as being appropriate to describe bariatric surgery procedures that form the basis for case ascertainment of data submitted to the NBSR for the annual outcome reporting. Codes that could potentially lead to incorrect inclusion of patients undergoing gastric surgery for non‐bariatric indications (such as malignancy/benign disease) were excluded. Codes and code combinations with a low incidence, and describing a primary bariatric procedure otherwise described by a more populous code, were excluded. Finally, codes listed as ‘other specified procedure’, ‘non‐specific procedure’ and adjuncts to primary bariatric procedures were also excluded.

The final list of 15 OPCS codes used to identify bariatric procedures, categorized by operation type, compared with the HSCIC codes used for previous analysis and currently used by NHS Digital, is shown in *Table* 1. The four operation types were: gastric bypass (any bypass including Roux‐en‐Y), implant/temporary (gastric band, balloon, bubble), sleeve gastrectomy (including this operation plus duodenal switch) and duodenal switch alone.

**Table 1:** OPCS codes utilized and excluded by the present study relative to the Health and Social Care Information Centre/NHS Digital codes used in previous outcome analysis

Bariatric procedure	Utilized codes*	Excluded codes*
Bypass	G281, G301, G302, G304, G312, G321, G331	G288, G289, G310, G311, G313, G314, G315, G316, G318, G319, G320, G322, G323, G324, G325, G328, G329, G330, G332, G333, G335, G336, G338, G339
Sleeve gastrectomy	G282, G283, G284, G285	
Implant/temporary	G303, G481, G485	G308, G309
Duodenal switch	G716	

* Codes and their full descriptors are shown in *Table* S1 (supporting information).

### Outcome measures

The main outcome measures were in‐hospital mortality and 30‐day mortality after discharge. For the purposes of the HES data set, in‐hospital mortality is defined as a death occurring within the primary admission and before discharge. However, any death occurring after discharge, even when associated with a postoperative complication, is not captured by this measure.

ONS 30‐day postdischarge mortality is defined as any death occurring within 30 days of discharge from the primary admission, and captures readmissions and subsequent postoperative deaths within 30 days.

### Statistical analysis

Counts of operations and mortality for each financial year from 2009–2010 to 2015–2016 were extracted from the HES database. Owing to NHS data governance reporting guidelines, exceedingly low mortality figures for a given year (fewer than 5) are not published, to avoid potential patient identification. Statistical analysis was conducted using SPSS^®^ version 22.0 (IBM, Armonk, New York, USA). The χ^2^ test was used to evaluate categorical variables. *P* < 0·050 was considered statistically significant.

## Results

### Procedural analysis: HES data set

Between 2009 and 2016, HES data indicated 41 241 primary bariatric procedures, a mean of 5892 procedures per year. In comparison, procedural counts using the code choice currently used by the HSCIC and in previous HES analysis^8^ (*Table* 1) identified 45 416 procedures over the same period. Operation subtype‐specific procedural counts are shown, indicating increased uptake of sleeve gastrectomy and a decreasing rate of gastric banding (*Table* 2).

**Table 2:** Annual and total procedure‐specific case counts over the study period

Year	Procedure			
	Bypass	Sleeve gastrectomy	Implant/temporary	Duodenal switch
2009–2010	3149	555	1649	10
2010–2011	3752	900	1544	15
2011–2012	3739	1410	1540	12
2012–2013	3672	1627	1110	8
2013–2014	3542	1544	704	18
2014–2015	3252	1764	524	17
2015–2016	2895	1777	494	18
Total	24 001	9577	7565	98

### Mortality analysis

*Table* 3 shows the annual HES in‐hospital and ONS 30‐day postdischarge mortality rates, both indicating a trend for decreasing mortality. In‐hospital mortality fell from eight deaths in 2009–2010 to six in 2013–2016 (*P* = 0·046).

**Table 3:**
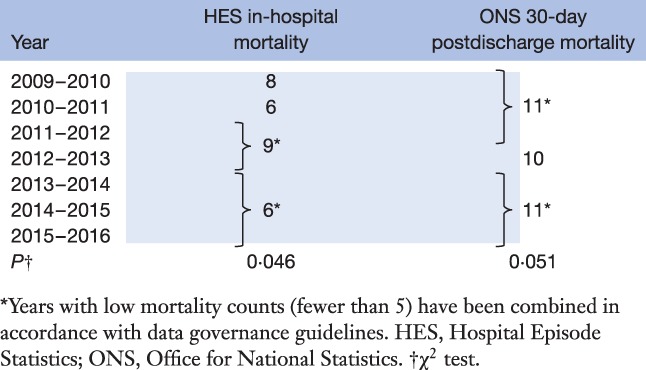
Annual in‐hospital (Hospital Episode Statistics) and 30‐day postdischarge (Office for National Statistics) deaths from primary bariatric surgery over the study period

The cumulative HES in‐hospital mortality rate was 0·07 per cent (29 of 41 241) and the ONS 30‐day postdischarge mortality rate was 0·08 per cent (32 of 41 241) (*Table* 4). In‐hospital and 30‐day mortality rates after discharge using codes employed in the previous mortality analysis^8^ (*Table* 1) are shown for comparison. There were no statistically significant differences in in‐hospital or 30‐day postdischarge mortality rates between the two sets of code choices (*Table* 4). Owing to the low number of total deaths, it was not possible to split mortality rates by procedure.

**Table 4:** Comparison of cumulative in‐hospital and 30‐day mortality rates after discharge determined using codes employed in the present study and codes used in previous mortality analysis

	Cumulative mortality measure	
	In‐hospital mortality (%)	30‐day postdischarge mortality (%)
Codes used in present study (*n* = 15)	0·07	0·08
Codes used in previous analysis (*n* = 37)	0·07	0·06
*P* *	0·824	0·190

* χ^2^ test.

## Discussion

Over the 7‐year study period, in‐hospital and 30‐day deaths from primary bariatric procedures in the NHS have remained low. The static in‐hospital and 30‐day mortality rates after discharge are encouraging, particularly given the reduction in temporary/implant procedures such as gastric banding, and the increased use of a more major intervention – sleeve gastrectomy^14^. Low mortality was independent of the choice of code used to identify bariatric procedures, with no significant difference between the mortality rates identified using a refined set of 15 codes in the present study and the 37 codes used currently by the HSCIC^8^ ^13^. There was a statistically significant trend for decreased in‐hospital mortality over the period of analysis, although, owing to the overall low number of deaths, caution needs to be exercised in interpreting this as implying progressive improvement.

The in‐hospital mortality rate of 0·07 per cent and 30‐day mortality rate of 0·08 per cent are similar to those reported by the UK NBSR. Between 2009 and 2016, the NBSR reported a UK‐wide in‐hospital mortality rate of 0·08 per cent (31 of 39 745) in two comprehensive registry reports and elsewhere^6^ ^12^, ^15^. Although HES data presented here are limited to mortality in England, the similarity between the present results and mortality data reported by the NBSR supports the accuracy of reporting, and suggests that HES data are representative of mortality across the UK.

Mortality in the present study is similar to that in other reports. Analysis of postoperative outcomes between 2005 and 2009 from ten centres (6118 patients) in the USA showed a mortality rate of 0·3 per cent within 30 days of surgery^16^. The Michigan Bariatric Surgery Collaborative study^17^ of 15 275 patients reported a 30‐day mortality rate of 0·14 per cent after gastric bypass. A more recent meta‐analysis^18^ of 164 studies from 2003 to 2012 reported a 30‐day mortality rate of 0·08 per cent in RCTs and 0·22 per cent in observational studies. Bariatric surgery is thus as safe, or safer, than many other common elective procedures^19^ ^20^.

In an earlier analysis of HES 30‐day mortality figures^8^, a mortality rate of 0·30 per cent was found following bariatric procedures conducted between 2000 and 2008, when 36 per cent of procedures were open operations. The present results showed fewer in‐hospital and 30‐day postdischarge deaths, despite a substantial increase in the annual number of primary bariatric procedures conducted since 2008. Although small in absolute terms, this difference may reflect improved techniques and the increased use of laparoscopic procedures, known to have better outcomes than those for open surgery^8^ ^21^. Although the present study did not assess the proportion done openly, laparoscopy is now standard in bariatric surgery, accounting for over 95 per cent of primary procedures recorded in the NBSR in 2011–2013^6^. Mortality rates for open *versus* laparoscopic approaches have not been reported from the NBSR.

The present study used 15 codes to identify and subsequently group the cohort into four types of bariatric procedure. In the earlier HES analysis^8^, 37 codes were used to define the three predominant bariatric procedures of gastric bypass, gastrectomy and gastric banding. The discrepancy in the number of codes included is due to the exclusion of 26 codes, and the subsequent inclusion of four additional codes (G284, G481, G485 and G716), through the methodology of code selection used in the present study, as indicated in *Table* 1. As the descriptors demonstrate (*Table* S1, supporting information), most of the excluded codes were either revisional procedures, adjuncts to primary surgery, or non‐specific/unspecified procedures. Inclusion of such codes may have resulted in the capture of non‐bariatric procedures, such as oesophagostomy, and revisional bariatric surgery, which may have different outcomes relative to those of primary bariatric procedures^22^ ^23^.

Although data capture in the NHS is based on HES, it is known to have potential inaccuracies^24^. Regarding surgical procedures, this can occur through either missing or misidentifying patients. For instance, the HSCIC recently down‐adjusted the annual volume of bariatric surgery by several hundred patients due to the recording of gastric band adjustments in the clinic at one hospital as separate procedures^25^. In comparison with the present results, the NBSR (at the time a voluntary registry) reported 1052 primary bariatric procedures in 2009–2010^6^. Such findings are likely to be sequelae of the current coding system. Although experienced and skilled, clinical coders assign codes based on their interpretation of the operative summary, notes or discharge letter^26^. This can lead to variability in the coding of similar procedures between coders and impact the case‐mix reported by HES^26^, ^27^, ^28^.

The process used to refine the plethora of potential bariatric codes to the 15 most relevant ones used in the present study highlights deficiencies in the current coding system. In the absence of linking data of actual patients between HES and the NBSR, investigators must rely on textual analysis of the code descriptions backed up with a survey of code usage to define a minimum set of bariatric codes, as used here. It is not feasible within data governance limitations to link actual patient data between the NBSR and HES. To prevent such coding variability and to ensure the HES database remains the standard, it is suggested that OPCS codes for bariatric surgery should be refined in accordance with the procedure descriptions in the NBSR Clinical Outcomes Reports. This needs greater input and involvement from bariatric surgeons. In support of this, for the 3 years from 2013 to 2016 the overall total case ascertainment for individual patient records submitted to the NBSR for NHS England was 92·5 per cent (20 534 of 22 199) of the HES‐recorded case volume^14^.

Bariatric surgery is associated with a low risk of procedure‐related death. The increased uptake of bariatric surgery in 2009–2016 within the English NHS has been safe. The perceived risk of bariatric surgery should not be seen as a barrier for severely obese patients seeking treatment.

**Supporting information**

Additional supporting information may be found online in the supporting information tab for this article.

## Supporting information


**Table S1** OPCS codes and associated descriptors used to identify patients undergoing bariatric surgery for the present study and those utilized previously but excluded from the present study
