# Supplementary material for: Mortality related to primary bariatric surgery in England
Source: BJS Open. 2017 Oct 26;1(4):122–7. doi: 10.1002/bjs5.20 (PMC5989948; doi:10.1002/bjs5.20)
Supplement: Supplementary file 1 — Table S1 OPCS codes and associated descriptors used to identify patients undergoing bariatric surgery for the present study and those utilized previously but excluded from the present study [file BJS5-1-122-s001.docx]

**BJS5_20**

**Mortality related to primary bariatric surgery in England**

**M. Alam, S. Bhanderi, J. H. Matthews, D. McNulty, D. Pagano, P. Small, R. Singhal and R. Welbourn**

**Table S1** OPCS codes and associated descriptors used to identify patients undergoing bariatric surgery for the present study and those utilized previously but excluded from the present study

| OPCS codes included in the present study | Procedure |
| --- | --- |
| G281 - Partial gastrectomy and anastomosis of stomach to duodenum | Gastric Bypass |
| G301 - Gastroplasty NEC |  |
| G302 - Partitioning of stomach NEC |  |
| G304 - Partitioning of stomach using staples |  |
| G312 - Bypass of stomach by anastomosis of stomach to duodenum |  |
| G321 - Bypass of stomach by anastomosis of stomach to transposed jejunum |  |
| G331 - Bypass of stomach by anastomosis of stomach to jejunum NEC |  |
| G282 - Partial gastrectomy and anastomosis of stomach to transposed jejunum | Sleeve Gastrectomy |
| G283 - Partial gastrectomy and anastomosis of stomach to jejunum NEC |  |
| G284 - Sleeve gastrectomy and duodenal switch |  |
| G285 – Sleeve gastrectomy NEC |  |
| G303 - Partitioning of stomach using band | Implant / Temporary |
| G481 - Insertion of gastric bubble |  |
| G485 - Insertion of gastric balloon |  |
| G716 - Duodenal switch | Duodenal switch |
| **OPCS codes excluded from the present study** | **Procedure** |
| G288 - Other specified partial excision of stomach | Gastric Bypass |
| G289 - Unspecified partial excision of stomach |  |
| G310 - Conversion of previous anastomosis of stomach to duodenum |  |
| G311 - Bypass of stomach by anastomosis of oesophagus to duodenum |  |
| G313 - Revision of anastomosis of stomach to duodenum | Gastric Bypass |
| G314 - Conversion to anastomosis of stomach to duodenum |  |
| G315 - Closure of connection of stomach to duodenum |  |
| G316 - Attention to connection of stomach to duodenum |  |
| G318 - Other specified connection of stomach to duodenum |  |
| G319 - Unspecified connection of stomach to duodenum |  |
| G320 - Conversion from previous anastomosis of stomach to transposed duodenum |  |
| G322 - Revision of anastomosis to transposed jejunum |  |
| G323 - Conversion to anastomosis of stomach to transposed jejunum |  |
| G324 - Closure of connection of stomach to transposed jejunum |  |
| G325 - Attention to connection of stomach to transposed jejunum |  |
| G328 - Other specified connection of stomach to transposed jejunum |  |
| G329 - Unspecified connection of stomach to transposed jejunum |  |
| G330 - Conversion from previous anastomosis of stomach to jejunum not elsewhere classified |  |
| G332 - Revision of anastomosis of stomach to jejunum not elsewhere classified |  |
| G333 - Conversion to anastomosis of stomach to jejunum not elsewhere classified |  |
| G335 - Closure of connection of stomach to jejunum NEC |  |
| G336 - Attention to connection of stomach to jejunum |  |
| G338 - Other specified connection of stomach to jejunum |  |
| G339 - Unspecified other connection of stomach to jejunum |  |
| G308 - Other specified plastic operations on the stomach | Gastric Banding |
| G309 - Unspecified plastic operations on the stomach |  |
